# An Ensemble Strategy to Predict Prognosis in Ovarian Cancer Based on Gene Modules

**DOI:** 10.3389/fgene.2019.00366

**Published:** 2019-04-24

**Authors:** Yi-Cheng Gao, Xiong-Hui Zhou, Wen Zhang

**Affiliations:** Hubei Key Laboratory of Agricultural Bioinformatics, College of Informatics, Huazhong Agricultural University, Wuhan, China

**Keywords:** prognosis gene, ovarian cancer, subtype, gene co-expression network, ensemble classifier

## Abstract

Due to the high heterogeneity and complexity of cancer, it is still a challenge to predict the prognosis of cancer patients. In this work, we used a clustering algorithm to divide patients into different subtypes in order to reduce the heterogeneity of the cancer patients in each subtype. Based on the hypothesis that the gene co-expression network may reveal relationships among genes, some communities in the network could influence the prognosis of cancer patients and all the prognosis-related communities could fully reveal the prognosis of cancer patients. To predict the prognosis for cancer patients in each subtype, we adopted an ensemble classifier based on the gene co-expression network of the corresponding subtype. Using the gene expression data of ovarian cancer patients in TCGA (The Cancer Genome Atlas), three subtypes were identified. Survival analysis showed that patients in different subtypes had different survival risks. Three ensemble classifiers were constructed for each subtype. Leave-one-out and independent validation showed that our method outperformed control and literature methods. Furthermore, the function annotation of the communities in each subtype showed that some communities were cancer-related. Finally, we found that the current drug targets can partially support our method.

## Introduction

Cancer is a disease that seriously endangers human health (Siegel et al., 2017). Cancer prognosis research is very important to avoid patients receiving excessive or improper treatment (Domany, 2014; Kourou et al., 2015). Ovarian cancer is one of the most common malignant tumors and there is an urgent need to develop new treatment methods to improve the prognosis (Wang et al., 2017). Identifying prognostic genes in cancer is important not only for the treatment of cancer patients but also for drug discovery (Wang et al., 2017). Therefore, the selection of prognostic genes and prognosis prediction for ovarian cancer is of great importance (Konecny et al., 2016).

These days, many methods have been used in solving biological problems by using high-throughput biological data (Zhang et al., 2017, 2018a,b,c,d,e,f) and machine learning algorithms (Zhang et al., 2008). However, the existing models for predicting the outcomes of ovarian cancer are poorly generalized (Konecny et al., 2016), possibly due to the high heterogeneity of cancer (Burrell et al., 2013). Even in the same cancer, it can be divided into different subtypes (Jiang et al., 2019), but most of the existing methods do not take this into account (Yu et al., 2016; Pawlovsky and Matsuhashi, 2017). Recent literature has confirmed that considering the subtype of cancer and then constructing the cancer prognosis model is conducive to the improvement of the performance of the cancer prognosis model (Yu et al., 2018).

In addition, cancer is a complex disease and the occurrence of cancer is usually not caused by a single gene, but by the combined action of multiple genes (Yang et al., 2014a). Many current prognostic methods do not take this into account (Petitjean et al., 2007; Hu et al., 2010). Gene co-expression networks are able to reflect the interrelationships between genes in biological processes (Guo et al., 2015; Deng et al., 2016; Serin et al., 2016). A community (dense cluster) in a biological network can work together as a basic functional module to participate in the occurrence of diseases (Zhou et al., 2014). Therefore, the community in the gene co-expression network in cancer patients may be related to the prognosis of cancer, and multiple communities related to the prognosis of cancer may more comprehensively reflect the prognosis process of cancer.

In this work, we first applied clustering analysis to the data set of ovarian cancer from TCGA (The Cancer Genome Atlas) (Network, 2008) in order to divide the patients into different subtypes. Our clustering results were validated using survival analysis to determine whether patients in different subtypes had different survival risks. We then constructed a co-expression network for each subtype. In this network, the correlation between genes was determined by measuring the Pearson's correlation coefficient (Sedgwick, 2012). Then, we mined the dense clusters as gene communities in each network (Ruan et al., 2010; Zhou et al., 2014). Based on the communities in each subtype, we construct an ensemble classifier to predict the cancer prognosis in the corresponding subtype. To validate the performance of our model, we compared it with two control models: the classifier constructed without clustering information and the classifier with clustering information but without the gene co-expression network. Furthermore, we also compared our method with two models based on the published papers. Finally, we adopted the functional annotation with these community modules in each subtype to reveal some biological mechanisms of cancer. In addition, based on these communities, we used hypergeometric distribution tests to validate whether these communities could be used to screen drugs for ovarian cancer.

## Materials and Methods

### Data Set and Preprocessing

To evaluate our method, two ovarian cancer data sets, each containing gene expression profiles and clinical information (including the time to death and the status of death) were collected in this work. One data set from TCGA (Network, 2008) containing 574 patients was used to test the model. A merged data set containing 1287 patients, collected from previous work (Gyorffy et al., 2012), was used as an independent data set. The platform of TCGA data set is Agilent G4502A. Since the merged data set contains the samples of TCGA, we removed the samples of TCGA and 782 samples were remained. Quantile normalization (Bolstad et al., 2003; Belorkar and Wong, 2016) was then applied to all the data sets in terms of data preprocessing. Since all the data sets come from gene chips, this standardized method can eliminate the errors caused by experimental technologies and keep the data of all samples at the same level (Bolstad et al., 2003).

The prognosis information of the cancer patients was discretized when constructing the classifier. If the death of a patient occurred within 3 years, we set the phenotype as high-risk. If a patient's total survival time was more than 3 years, we set the phenotype as low-risk. Otherwise, the patients that were alive but still within 3 years were abandoned.

In order to validate whether the genes involved in the community could be used to screen drugs for ovarian cancer patients, we obtained the drug target information from the Therapeutic Target Database (TTD) (Yang et al., 2015), DrugBank (Wishart et al., 2008; Law et al., 2014), and Drug-Gene Interaction Database (DGIdb) (Wagner et al., 2016). The drug targets were set as the combined set of the entire three databases. Adaptation diseases for each drug were also obtained from the three databases.

### Clustering Analysis

We applied the K-means (Jain, 2010) algorithm to cluster the cancer patients into different subtypes. First, the top 15% of genes with the greatest variance were selected as the clustering features as they are considered to contribute to interesting variance (Belorkar and Wong, 2016). Second, using the selected genes as features, we used the K-means (Jain, 2010) clustering method to divide the patients in TCGA into different subtypes, and the Euclidean distance was used to measure the distance between samples. Third, the Dunn Index (Dunn, 1973) was used as the indicator to evaluate the quality of the cluster and to find the best number of clusters, which are calculated from the following equation,

(1) $$\begin{array}{r} DI_{m}=\frac{\underset{1\leq i\leq j\leq m}{\min}\delta \left(C_{i},C_{j}\right)}{\underset{1\leq k\leq m}{\max}\Delta_{k}} \end{array}$$

where *m* is the number of clusters, Δ_*k*_ is the mean distance between all sample pairs in the same cluster *C*_*k*_, and δ(*C*_*i*_, *C*_*j*_) is the distance between the centroids of cluster *C*_*i*_ and *C*_*j*_. The higher the value of *DI*_*m*_, the better the quality of the cluster. Finally, we selected *m* with the highest Dunn Index as the number of subtypes.

### Constructing the Co-expression Network

In this work, we constructed a gene co-expression network for each subtype based on the gene expression data of cancer patients. First, the Pearson correlation coefficient was used to calculate the correlation between every two genes (Sedgwick, 2012). The Pearson correlation coefficient (*r*) was calculated as follows:

(2) $$\begin{array}{r} \text{r}=\frac{1}{n-1}\sum_{i=1}^{n}\left(\frac{X_{i}-\bar{X}}{\sigma_{X}}\right)\left(\frac{Y_{i}-Ȳ}{\sigma_{Y}}\right) \end{array}$$

where *n* is the number of cancer samples, *X* represents the gene expression value of gene *X* in sample *i*, $\bar{X}$ is the mean value of gene *X* in all samples, and σ_*X*_ is the standard deviation of gene *X* in all samples. Similarly, the values *Y*, $\bar{Y}$, and σ_*Y*_ correspond to the *Y* chromosome in the gene pairs.

Next, a rank-based method (Ruan et al., 2010) was applied to construct the gene co-expression network (Serin et al., 2016). For each gene, the top *n* genes most related to it were selected as its neighbors. In our project, we set *n* as 4 following a previous literature report (Ruan et al., 2010). Finally, all the selected gene pairs could create a co-expression network for each subtype.

### Network Visualization and Community Mining

Cytoscape 3.6.1 was used to visualize the network of every subtype and topology analysis was applied to these networks. The MCODE plug-in (Bader and Hogue, 2003) was then used in Cytoscape to mine communities in these networks.

### Constructing the Ensemble Classifier

Ensemble strategy has made great achievements in bioinformatics (Lin et al., 2013, 2014; Zhou et al., 2013; Zou et al., 2013, 2015; Wan et al., 2017). In this work, we also used an ensemble classifier to predict the prognostic of ovarian cancer patients. The main frame of constructing our ensemble classifier is shown in Figure S1. To begin with, our training data set was divided into different subtypes (Method 2.2) and we constructed the gene co-expression network for each subtype (Method 2.3). Then we mined the dense clusters as modules for each network (Method 2.4) and constructed the centroid classifier for each module as sub-classifier. In each subtype, the sub-classifiers were filtered by ACC (accuracy) and the ensemble classifier was constructed. The subtype of each sample in independent data set was determined and its prognosis was predicted by the corresponding ensemble classifier. The detail process to construct the prognostic model was shown as follow:

1. Based on the gene expression data, the subtypes of the ovarian cancer patients were identified by clustering analysis.
2. Using the gene expression profiles of the patients in each subtype, a gene co-expression network was constructed for each subtype.
3. The dense clusters in each gene co-expression network were detected.
4. The community modules in each subtype may have a significant effect on the prognosis of its corresponding subtype. Therefore, the genes involved in a community may be good features to predict the prognosis of cancer patients. In this work, the genes in each community were used as features to construct a classifier to predict the prognosis of cancer patients in the corresponding subtype. The centroid classifier is particularly suited for microarray data, which has large feature size but few samples. In addition, it has few model parameters and can even give better results than conventional methods (Abraham et al., 2010). Therefore, the centroid classifier was applied to construct the classifiers.
5. After that, the classifiers with an ACC (evaluated by leave-one-out validation) no less than a threshold were set as sub-classifiers and all the sub-classifiers were integrated as an ensemble classifier by majority voting strategy. Then an ensemble classifier was constructed for each subtype and the prognosis of each patient was predicted by the ensemble classifier of the corresponding subtype.

### Comparison With the Control Classifiers

In order to evaluate our main hypothesis that the clustering information and ensemble classifier based on communities in gene co-expression network could contribute to the prognosis of cancer patients, we compared our method with two controls. That is, the classifier without using the subtype information and the ensemble strategy, and the classifier using subtype information but not the ensemble strategy.

In the first control method, a centroid classifier is constructed without the subtype information and the gene co-expression network. The *t*-test is used to select the differentially expressed genes between low- and high-risk groups in all the patients. The *t*-test is calculated using the following equation (3),

(3) $$\begin{array}{r} \text{t}=\frac{\bar{X}-Ȳ}{\sqrt{\frac{S_{x}^{2}}{n}+\frac{S_{y}^{2}}{m}}} \end{array}$$

where $\bar{X}$ and $\bar{Y}$ are the average gene expression levels of low- and high-risk patients, respectively. *S*_*x*_ and *S*_*y*_ are their corresponding standard deviations, and *n* and *m* are the sizes of the data sets of patients with good and bad prognosis, respectively.

Based on the t-test, the top *n* genes with the smallest *p*-value were selected as features. We varied *n* from 1 to 100 and using the leave-one-out method each feature set was used to construct a centroid classifier to predict the prognosis in training data set. Next, we chose the control classifier with the best validation result as the final classifier. That is, only one classifier was construct for prognosis. The prognosis of the samples in independent data set were predicted directly using the chosen classifier.

The second control method used the clustering information to the construction of the model. That is, it constructed a centroid classifier for each subtype, and each centroid classifier was constructed using the same strategy with the first control method. For each patient in the independent data set, its subtype was determined based the Euclidean distance of the vector of its expression levels to the centroid of each subtype. That is, the patient was assigned to the subtype which it is the most similar with. And its prognosis was predicted using the centroid classifier of the corresponding subtype.

### Construction of the Representative Classifiers

In previous works, some gene-signatures were selected to predict the prognosis of ovarian cancer patients (Gyorffy et al., 2012; Martinez-Ledesma et al., 2015). Herein, we also compare our work with two literature methods (Gyorffy et al., 2012; Martinez-Ledesma et al., 2015).

The first method (Martinez-Ledesma et al., 2015) used 41 genes for cancer prognosis and the authors demonstrated that it could perform well on 11 types of cancer prognosis, including ovarian cancer. Using these genes, we constructed a centroid classifier based on a training data set which was denoted as a 38-gene classifier in this work (only 38 genes was present in our training data set).

The second method (Gyorffy et al., 2012) identified 34 genes which were considered to be related to the prognosis of ovarian cancer. Among the 34 genes, 33 were present in our training data set. Based on these genes, a centroid classifier was constructed (denoted as a 33-gene classifier in this work).

In their previous work, they both used the Cox model to evaluate their methods. Based on their gene signatures, we also used the Cox model to evaluate the prognostic capability of their genes. First, the Cox proportional hazards regression was applied to the correlation between each gene expression level and the prognostic risks of all the patients in TCGA. Next, we adopted the same strategy using the Gene expression Grade Index (Sotiriou et al., 2006) to calculate the prognosis risk of each patient in the independent data set, based on all the genes in the corresponding gene signature.

The risk score is calculated by the following equation (4),

(4) $$\begin{array}{r} \text{Risk Score}=\sum x_{i}-\sum y_{j} \end{array}$$

where x_*i*_ is the expression level of the gene whose Cox coefficient is positive and *y*_*i*_ is the expression level of the gene whose Cox coefficient is negative. According to the risk scores of the patients, they were equally divided into high- and low-risk groups.

### Performance Measures

The area under the curve (AUC), Matthews Correlation Coefficient (MCC) and Accuracy (ACC) were used as indexes to evaluate the classifiers in our work. The receiver operating characteristic (ROC) curve is a graphical plot that illustrates the sensitivity vs. one minus the specificity at different threshold settings. The AUC is the area under the curve and it is a widely used indicator to evaluate the performance of a classifier. The MCC is also an important indicator of the quality of classifiers and was used as an accuracy index in the US FDA-led initiative MAQC-II (Jurman et al., 2012). The MCC values fluctuate between −1 and +1 (a coefficient of +1 for completely correct predictions, 0 for meaningless predictions, and −1 for completely incorrect predictions) (Zhou et al., 2012). The ACC is the most natural performance measure indicator (Jurman et al., 2012). It is defined as the probability that a random event will be correctly classified, which is estimated by diving the number of correct classes by the total number of classes (Klinkenberg and Renz, 1998).

### Enrichment Analysis

We used the Gene Set Enrichment Analysis (GSEA) (Subramanian et al., 2005) to perform functional annotation of the genes in the selected communities of each subtype. The hypergeometric distribution test (5) was used to calculate whether the intersection set between the genes in a community and the targets of a drug were significant:

(5) $$\begin{array}{r} \text{p}-\text{value}=1-\sum_{i=0}^{m-1}\frac{\left(\begin{array}{c} M\\ i \end{array}\right)\left(\begin{array}{c} N-M\\ n-i \end{array}\right)}{\left(\begin{array}{c} N\\ n \end{array}\right)} \end{array}$$

where *N* is the number of all the genes in our training data set, *M* is the number of genes in the community, *n* is the number of the targets of the drug, and *m* is the size of the intersection set. The hypergeometric distribution test was also used to test whether the ratio of screened cancer drugs by the community is significantly high, compared with the number of cancer drugs in the entire database.

## Result

### Survival Analysis of the Cancer Patients in the Three Subtypes

**Figure 1:**
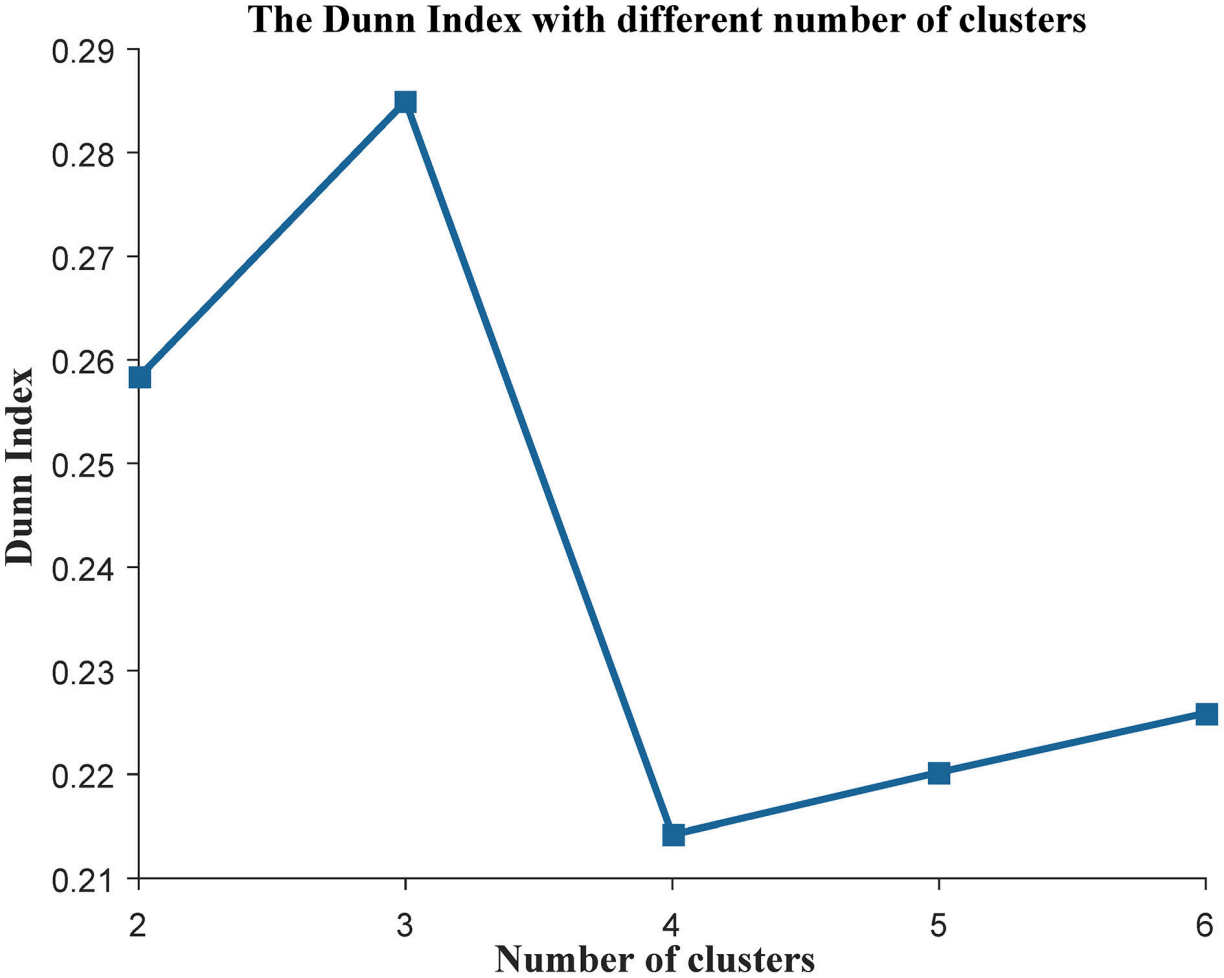
The evaluation of the clustering analysis in the training data set. The Dunn Index was used to evaluate the quality of the clustering analysis. The K (the number of the clusters) was varied from 2 to 6.

**Figure 2:**
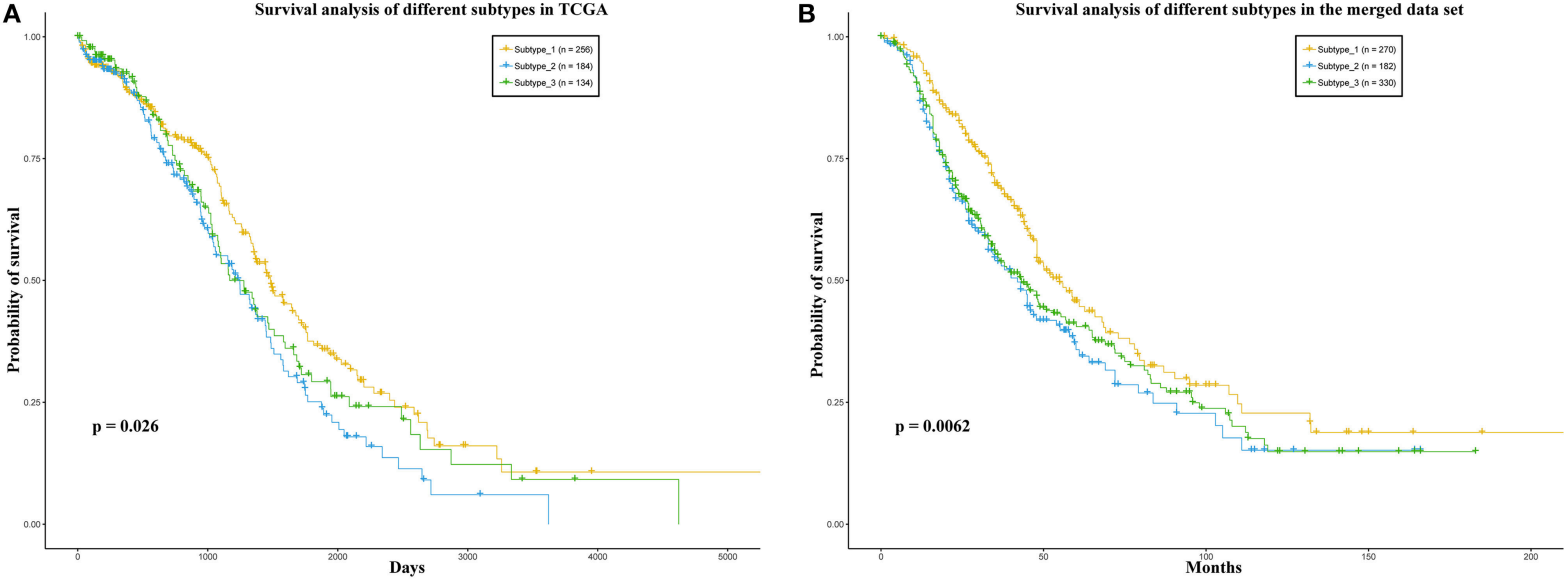
**(A)** TCGA, **(B)** the merged data set. Survival analysis of the patients in the three sub-types. In each data set, the patients were divided into three groups according to the three sub-types.

Some works have succeeded in identifying the subtypes of cancer patients based the high—throughput data of cancer patients (Sørlie, 2004; Justin et al., 2015; Jiang et al., 2019). In order to reduce the heterogeneity among the patients in the same group, we divided the ovarian cancer patients into different subtypes based on transcriptome data of these patients. In this work, we used K-means to cluster the patients into different subtypes. The Dunn Index (Dunn, 1973) was used to evaluate the clustering quality on the TCGA training data set, where the number of clusters (K) was varied from 2 to 6. The Dunn Index of the clustering result shows that the optimal number of clusters is three (Figure 1). In addition, we applied survival analysis to patients in the three subtypes of the TCGA data set, which indicated that the patients in our three subtypes have different survival risks (Figure 2A). In a previous work, NMF clustering method was applied to cluster the ovarian cancer patients into four subtypes (Network, 2011). Here, we also applied this method to the training data set and survival analysis shows that the difference of the survival risks of the patients divided by our method is slightly significant than theirs (Figure S2). Considering that our approach is simpler, we still use K-means to cluster the cancer patients in ovarian cancer.

In addition, we also divided the patients in the merged data set into three subtypes according to the Euclidean distance between the expression level vector of each patient in the independent data set and the centroid of the clusters in training data set. We also applied survival analysis to the patients in the three subtypes on the merged data set. As a result, the patients could be significantly distinguished by the survival probability (Figure 2B). These results may indicate that the three subtypes identified by our method have different prognostic risks and the patients in each subtype may have more similarities than the patients in different subtypes.

### The Co-expression Networks of the Three Subtypes

In order to describe the relationship among the genes in each subtype of ovarian cancer patients, we constructed a gene co-expression network for each subtype. Adopting a similar strategy as the rank-based method for each gene (Ruan et al., 2010), we selected the top four genes that were most related to its neighbors to construct the co-expression network. Each of the three networks has 11,049 nodes and 44,196 edges (Figure S3). The average number of neighbors in the network of the first, second and third subtype are 6.633, 6.617, and 6.525, respectively. All the three networks are shown in the Tables S1–S3. Furthermore, we applied the power-law fitting to the three networks, and the correlation and R-square of the fittings indicated that all the networks fitted the power law distribution well (Table 1). Our topology analysis showed that the three networks were scale-free and could be used to mine communities, which could be used to construct prognostic models in ovarian cancer.

**Table 1:** Correlation and R-square of power-law fitting in three networks.

**Networks**	**Correlation**	**R-square**
First subtype	0.836	0.948
Second subtype	0.860	0.940
Third subtype	0.778	0.929

### Forecasting Ability of Our Classifier

**Figure 3:**
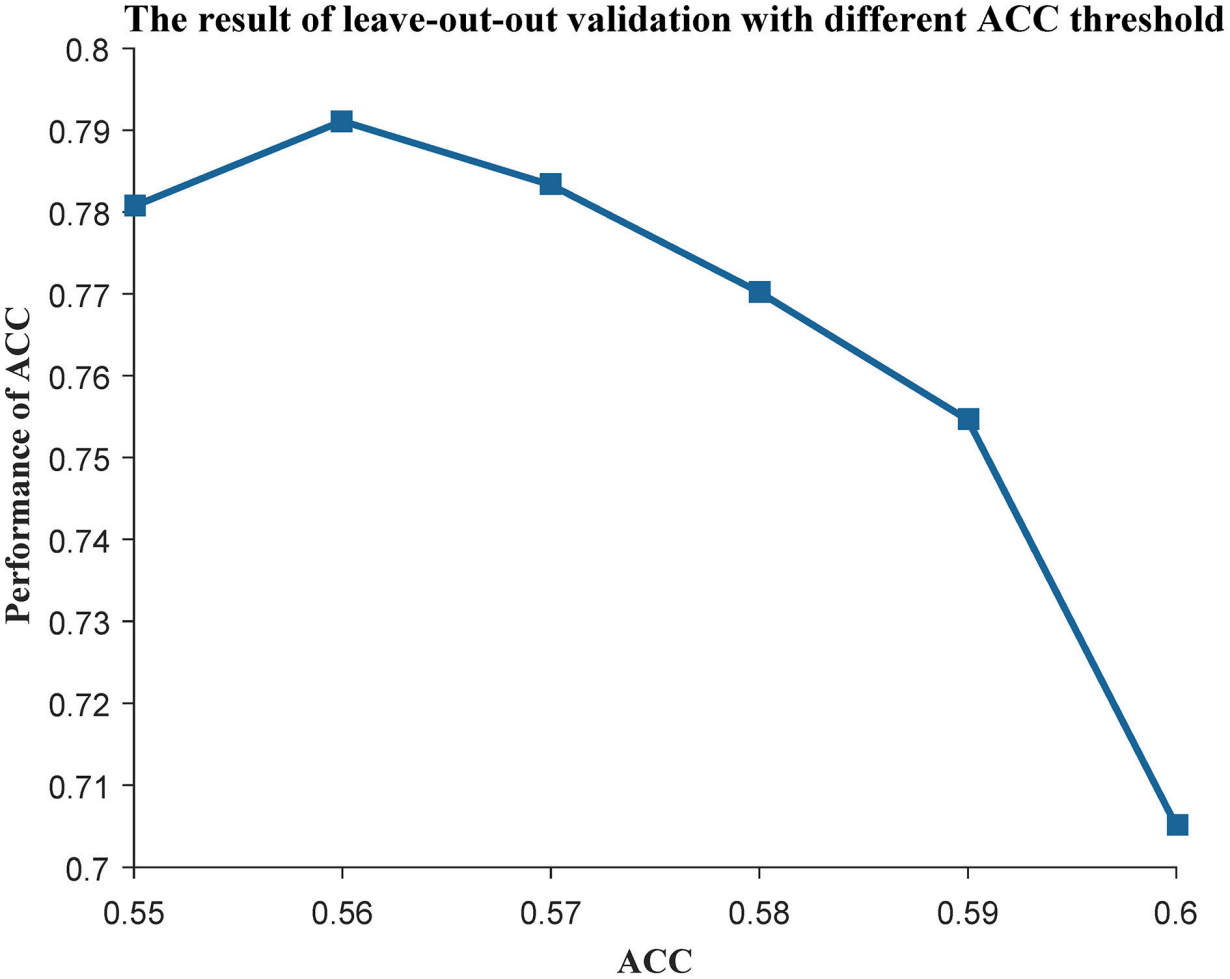
The leave-one-out result with different ACC threshold. The threshold of ACC was varied from 0.55 to 0.60.

As the genes in a community work together to play an important role in many biological processes, we used MCODE (Bader and Hogue, 2003) to mine the communities in each subtype. Next, we used the genes in each community as features to construct a centroid classifier to predict the prognosis of the cancer patients in the corresponding subtype, using leave-one-out validation to evaluate its performance. Using the majority voting strategy, the classifiers capable of distinguishing prognosis were selected as weak classifiers to construct the ensemble classifier. We used the ACC of the classifier as an index to evaluate its prognostic capability and changed the threshold from 0.55 to 0.60. As a result, the ensemble classifier based on the weak classifiers with an ACC of more than 0.56 could achieve the best performance (Figure 3). Finally, we obtained 50 communities in the first subtype (Table S4), 73 communities in the second subtype (Table S5), and 92 communities in the third subtype (Table S6). These subtypes can be used to construct three ensemble classifiers for the three subtypes, which could be used as prognostic models for ovarian cancer patients.

**Figure 4:**
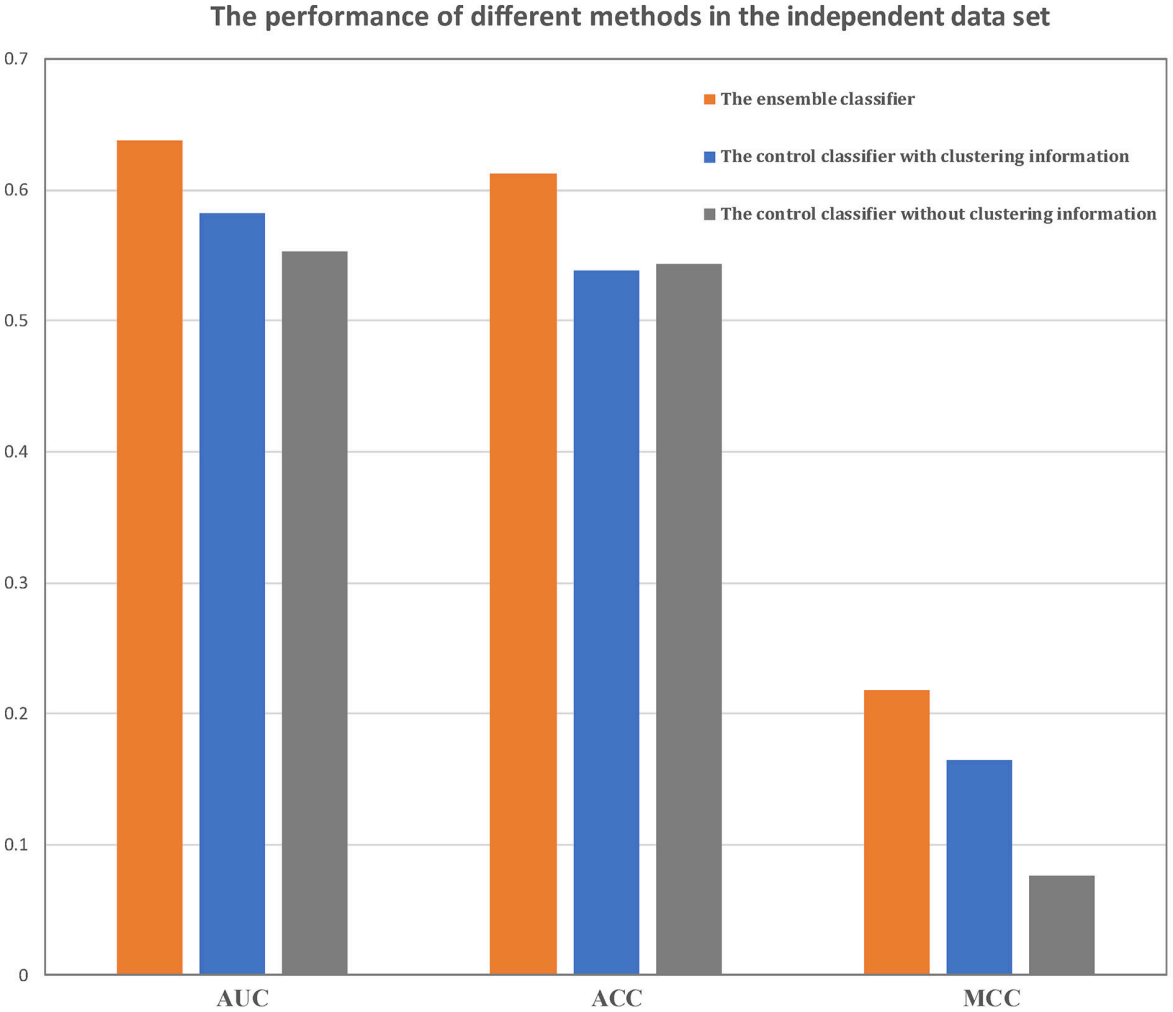
Comparison of the ensemble classifier with two control classifiers. The AUC, ACC and MCC of our classifier and two control classifiers on the independent data set.

In training data set, our method could achieve an AUC of more than 0.86, MCC of 0.57 and ACC of 0.79. An independent data set containing 782 samples was applied to verify our method. The AUC, MCC, and ACC values using our method were 0.64, 0.22, and 0.61, respectively (Figure 4). These performances showed that our classifier has a good forecasting ability in both the training and the independent data set.

### Comparison With Two Control Classifiers

In order to validate the hypothesis that the clustering information and the ensemble strategy based on gene co-expression network could improve the performance of the prognostic model, we compared the performance of our method with two control classifiers. The first one used a *t*-test to select features in all the patients in the training data set and used the selected features to construct a centroid classifier to predict the prognosis of all the ovarian cancer patients. The second one also used a *t*-test to select features and adopted the centroid classifier as a prognostic model but it constructed a centroid classifier for each subtype, i.e., the second method used clustering information in the process of constructing the model.

The performances of the two control methods in the training data set are shown in Table 2. From these results, it is evident that our method was better than both control methods, and the method without the clustering information is better than the method using clustering information. In addition, our classifier and the other two classifiers were independently verified using the independent data set (Figure 4). Our classifier can achieve an AUC of 0.64 (MCC of 0.22 and ACC of 0.61), the control classifier with clustering information had an AUC of 0.58 (MCC of 0.16 and ACC of 0.54), and the control classifier without clustering information had an AUC of 0.55 (MCC of 0.07 and ACC of 0.54).

**Table 2:** The leave-one-out result of classifier based on different methods.

	**Control classifier without clustering information**	**Control classifier with clustering information**	**Ensemble classifier**
AUC	0.6800	0.5517	0.8660
MCC	0.2986	0.0843	0.5727
ACC	0.658	0.5509	0.7911

Our method outperformed the two control methods in both the training data set and the independent data set. The control method with clustering information performed better in the independent data set, although it is not better in the training data set. As we know, the control method without clustering used all the samples in the training data set to construct the classifier. However, the control method with the clustering information only applied the samples in each subtype to fit the model. The classifier with more samples may perform better in the training data set. However, the independent data set does not perform well using this method because of overfitting, which may be caused by the high heterogeneity of the cancer patients in different subtypes.

As we know, the cox regression is also a frequently-used method to select features in cancer prognosis. Here, we also used cox regression to select features for the two control classifiers. A similar result could be found that our ensemble classifier was the best, and the control classifier using subtype information was better than the one without the subtype information (Figure S4). All these results showed that the clustering information and the ensemble strategy based on gene co-expression network could improve the performance of the prognostic model.

### Comparison With Two Representative Works

Two representative methods were compared with our method. We constructed the centroid classifiers based on their gene sets as we did in our work. From Figure S5, our classifier was better than the 33-gene and 38-gene classifiers. The AUC performance of our classifier achieved 0.64 and MCC achieved 0.22 (ACC of 0.61). Compared to our method, the other two classifiers AUC was lower and the MCC was 0 (ACC < 0.5). Thus, the classifier based on our method outperformed the other two methods.

**Figure 5:**
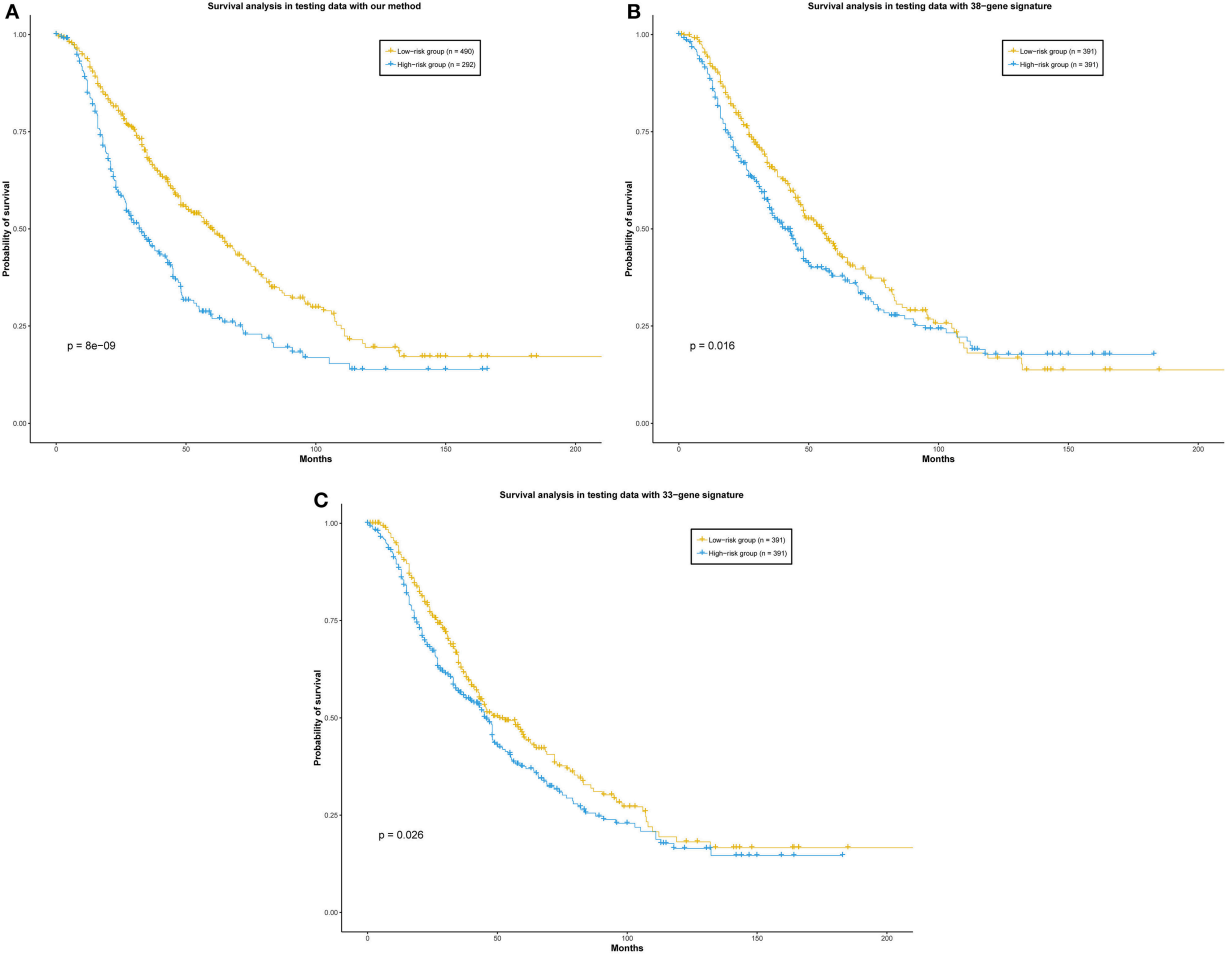
**(A)** Our method, **(B)** 38-gene signature, **(C)** 33-gene signature. Survival analysis of the patients divided by our ensemble classifier and representative works. Patients in the independent data set were divided into two groups according to the prediction result of our ensemble classifier, and the risk scores calculated by the representative gene sets, respectively.

In their previous work, they both used Cox models to evaluate their methods. Based on their gene signatures, we also used the Cox model to evaluate the prognostic capability of their genes (see Materials and Methods). In order to give a more direct comparison of the performance of our method with two other prognostic gene sets, a total of 789 patients of the merged data set were predicted prognostic outcome by our ensemble classifier. Meanwhile, their risk scores based on two gene sets from these two representative methods were calculated. As a result, the log-rank *p*-value between the patients in the two groups predicted by our method is 8 × 10^−9^. The *p*-values of the log-rank test between the low-risk and high-risk group calculated by the two representative methods are 0.016 and 0.026, respectively (Figure 5). In summary, our ensemble classifier outperformed the two representative methods, both in classification and survival analysis.

### Functional Annotation of the Filtered Communities

As the communities with distinguishing capability in cancer prognosis may play important roles in cancer prognosis, we applied enrichment analysis to the genes in the top ten communities according to the ACC performance in each subtype with gene ontology (GO) terms by GSEA (Subramanian et al., 2005). In these top ten communities, we selected the most significant related biological processes for each annotated community, which are listed in Tables S7–S9.

In the first subtype, three out of ten communities were significantly annotated. The first community was enriched by “ethanol metabolism process.” As we know, this biological process can produce a type of carcinogens-reactive aldehydes (Kottemann and Smogorzewska, 2013). In addition, “positive regulation of proteolysis” was reported to be related to the occurrence of ovarian cancer (Lengyel, 2010) and it was significantly enriched in the fourth community. In addition, “the glutathione derivative metabolic process” is the most significant one in the ninth community of the first subtype, with a *p*-value of 9.52 × 10^−11^. It was reported that glutathione played an important role in cancer progression and chemoresistance (Traverso et al., 2013).

In the second subtype, five communities were significantly enriched. Among them, disturbing “DNA metabolic process” was reported to contribute to oncogenesis (Hoeijmakers, 2001). In addition, the other four GO Terms were also significantly enriched, such as the “response to steroid hormone,” “response to endogenous stimulus,” “response to topologically incorrect protein,” and “response to fatty acid.” The steroid hormone receptor has been previously demonstrated to be a potential prognostic marker for ovarian cancer patient survival (Lenhard et al., 2012). The endogenous stimulus comes from the microenvironment difference between normal and tumor tissues, and it could be used to treat cancer (Yang et al., 2014b). The incorrectly folded protein could affect the survival of tumor cells (Goloudina et al., 2012), the fatty acid which had been validated to be related to the rapid growth of tumor (Nieman et al., 2011) and the abnormal expression of its synthase have been often found in ovarian cancer with poor prognosis (Kuhajda, 2000). From the results of the survival analysis of the patients in the three subtypes, the prognosis of the patients in the second subtype was the poorest.

In the third subtype, “protein localization to centrosome” and “cell cycle process” were significantly related to the sixth and the tenth community, respectively. The “protein localization to centrosome” demonstrated that some proteins could affect the tumor cell cycle by the centrosome (Zhou et al., 1998; Kimura et al., 1999) and that cell cycle proteins are promising targets in cancer therapy (Otto and Sicinski, 2017). In other words, the communities in the third subtype were annotated by two cell-cycle related GO Terms. To summarize, the communities in the three subtypes were all cancer-related but each different subtype corresponded to aspects of different biological processes.

### Drug Screening Using Filtered Communities

As described above, some communities in the three subtypes are cancer-related. Therefore, genes involved in these communities may be candidates for therapy. In this work, we used these communities to screen drugs using the hypergeometric distribution test. We tested whether the targets for each drug could be enriched significantly with the genes in the corresponding community (Materials and methods).

In the first subtype, three drugs were screened by the community which was annotated as “positive regulation of proteolysis.” Among these three drugs, two drugs could be used as therapy for ovarian cancer. They were Carfilzomib (Tagawa et al., 2012) and Bortezomib separately and both could target to the gene PSMB1 in this community. Specially, Bortezomib has been used as the treatment drug for ovarian cancer in clinical trials (Bruning et al., 2009). The ratio of drugs that could be used as therapy for ovarian cancer, among the drugs screened by the community, is significantly high compared with the proportion of the ovarian cancer drugs among all the drugs in the database, with a *p*-value of 0.021. In the second subtype, nine drugs were obtained by the community (enriched by the “DNA metabolic process”) and six drugs could be used to treat ovarian cancer, and the *p*-values of the community were 2.73 × 10^−5^. Specially, the drug Niraparib could target to the PARP2 in this community and it was one of the most familiar drug for recurrent ovarian cancer (Kanjanapan et al., 2017; Scott, 2017). Using the community enriched by the “response to endogenous stimulus,” 183 drugs were screened and 115 drugs could be used for cancer patients, (*p*-value of 4.08 × 10^−7^). In the third subtype, the ratio of drugs for ovarian cancer screened by the community, which were related to the “cell cycle process,” was significantly higher than the ratio in all the drugs (*p*-value of 2.32 × 10^−4^). Among the 50 drugs screened by the community, 13 drugs were used as therapy for ovarian cancer and all of the drug could target to the YES1 or TYMS. Among these drugs, the Dasatinib could inhibit YES1 directly (Pathak et al., 2015). Besides, the drug Gemcitabine is reported that it can combine the gene TYMS to regulate the cell cycle (Duran et al., 2017). All these results indicate that the genes involved in the filter communities may be candidates for drug targets in ovarian cancer.

## Conclusion

Considering the heterogeneity and complexity of ovarian cancer, we demonstrated a new method to predict the prognosis of ovarian cancer based on the clustering information and gene co-expression network in each subtype of cancer patients. We divided the ovarian cancer data into three subtypes by clustering analysis and we found that the survival risks in these three subtypes were significantly different. We mined the important communities based on the co-expression networks in each subtype. There are 50, 73, and 92 communities in the first, second and third subtype, respectively. Next, we constructed a new ensemble classifier based on these communities to predict the prognosis of cancer. Compared to other literature methods, our classifier had improved performance. Furthermore, the function annotation of the communities in each subtype showed that some representative communities were cancer-related and the enrichment analysis of the genes in the communities with the drug-ontology data can partially support our biomarker identification method.

## Author Contributions

X-HZ and WZ designed the research. X-HZ and Y-CG performed the research and wrote the paper. Y-CG analyzed the data. All authors revised the manuscript.

### Conflict of Interest Statement

The authors declare that the research was conducted in the absence of any commercial or financial relationships that could be construed as a potential conflict of interest.
