# Supplementary material for: An Ensemble Strategy to Predict Prognosis in Ovarian Cancer Based on Gene Modules
Source: Front Genet. 2019 Apr 24;10:366. doi: 10.3389/fgene.2019.00366 (PMC6491874; doi:10.3389/fgene.2019.00366)
Supplement: Supplementary file 10 [file Data_Sheet_1.docx]

Supplementary Material

An ensemble strategy to predict prognosis in ovarian cancer based on gene modules

# Supplementary Tables

There are nine tables in the supplementary. And all of these tables are separate files. The titles of these tables are shown as follow: Table S1: The co-expression network of Subtype_1, Table S2: The co-expression network of Subtype_2, Table S3: The co-expression network of Subtype_3, Table S4: The filtered communities in Subtype_1, Table S5: The filtered communities in Subtype_2, Table S6: The filtered communities in Subtype_3. Table S7: Functional annotation of the first subtype, Table S8: Functional annotation of the second subtype, Table S9: Functional annotation of the third subtype.

# Supplementary Figures

There are five figures in the supplementary. The title of this figure as follow:


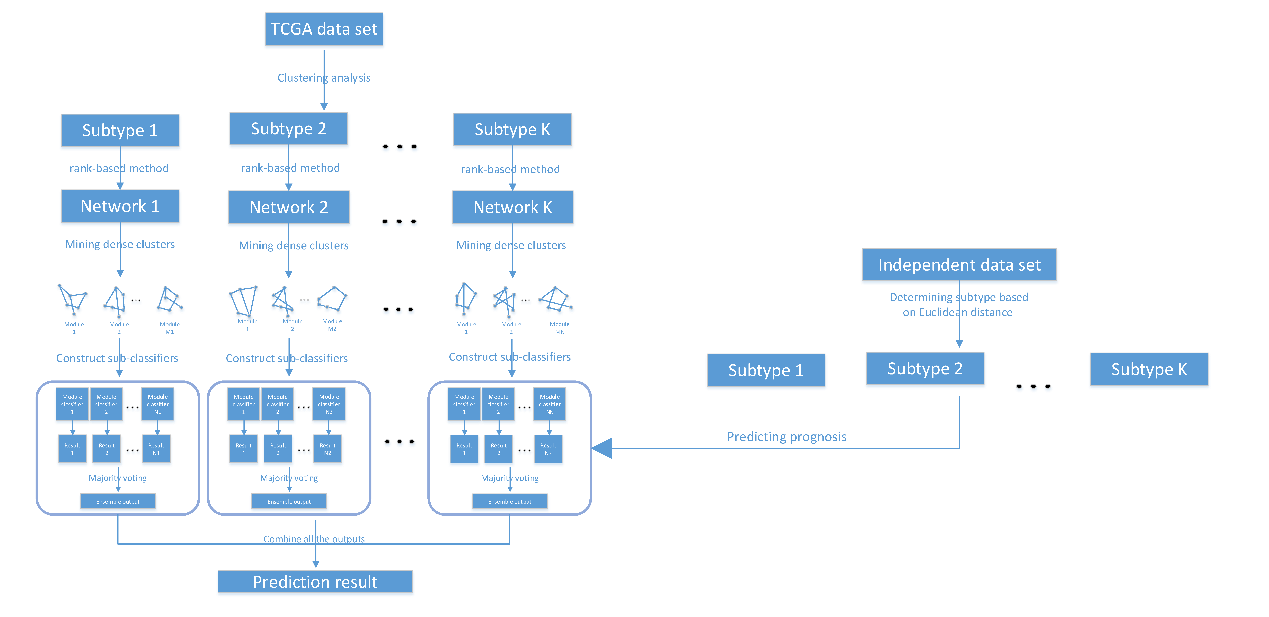


Figure S1: Workflow for constructing the ensemble classifier. The main frame of our work.


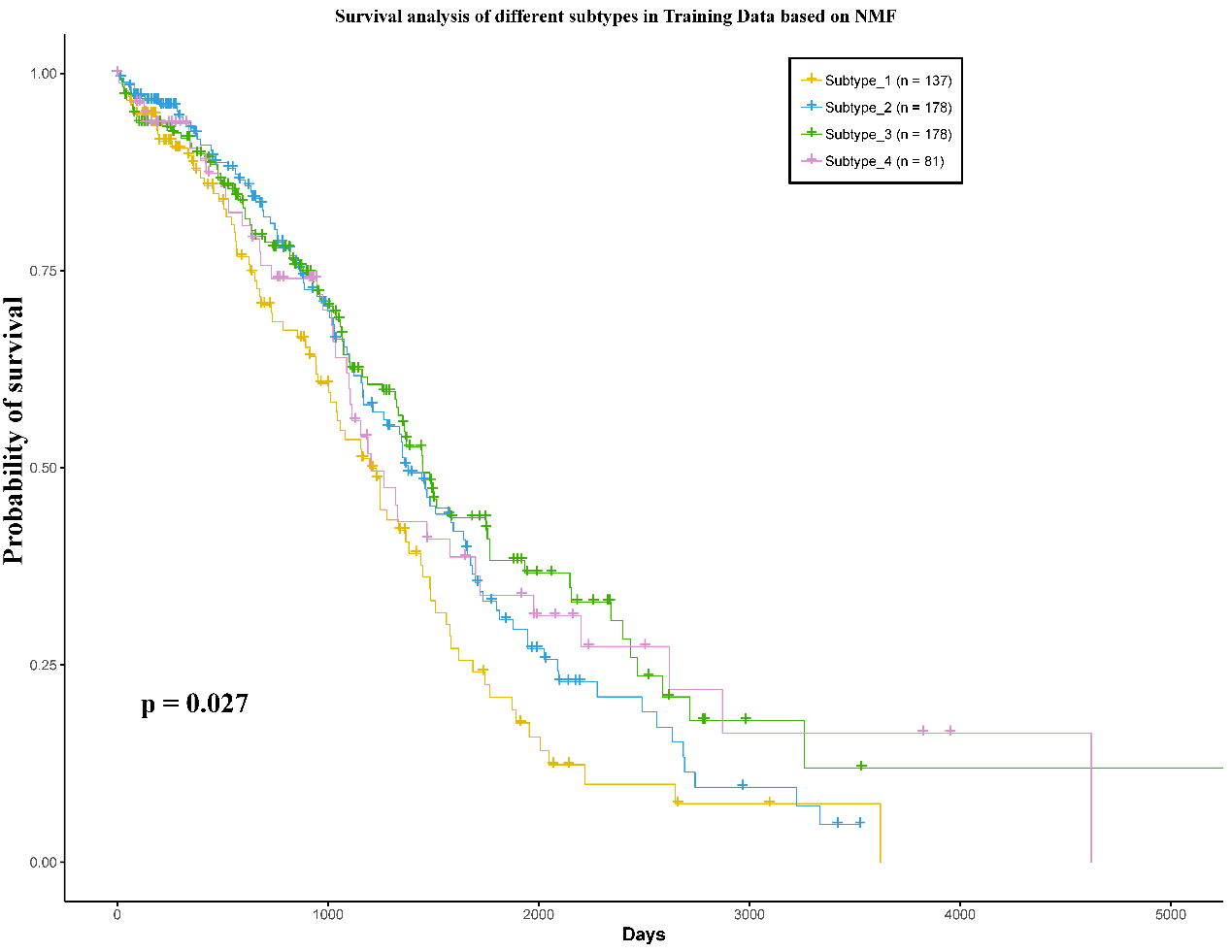


Figure S2: Survival analysis of different subtypes in TCGA with the NMF clustering method.


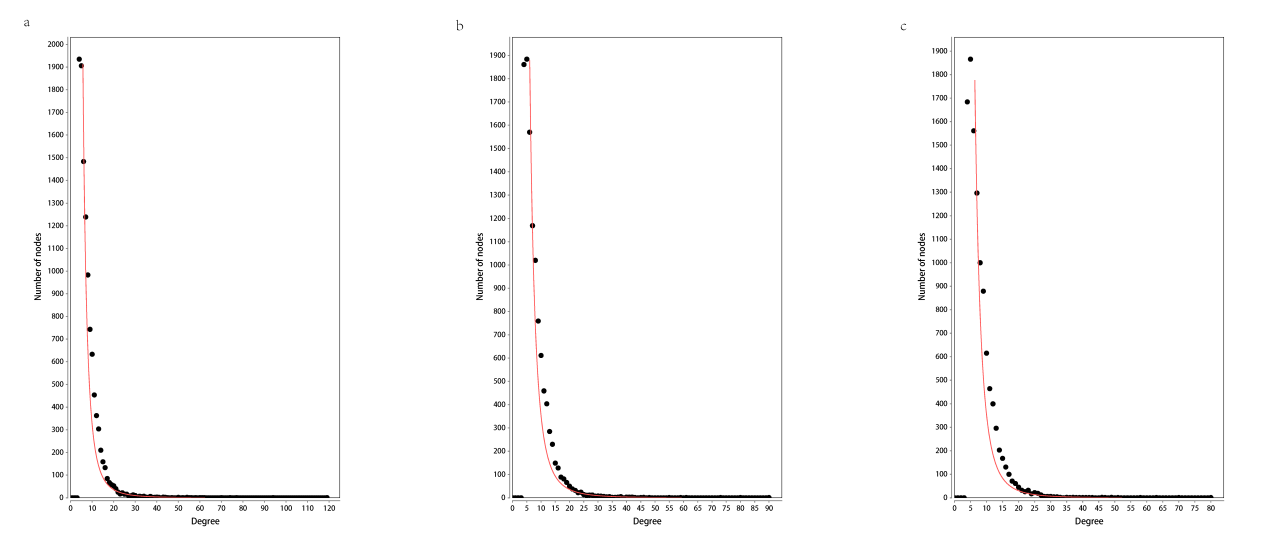


Figure S3: the power law-fit of the three sub-type networks.


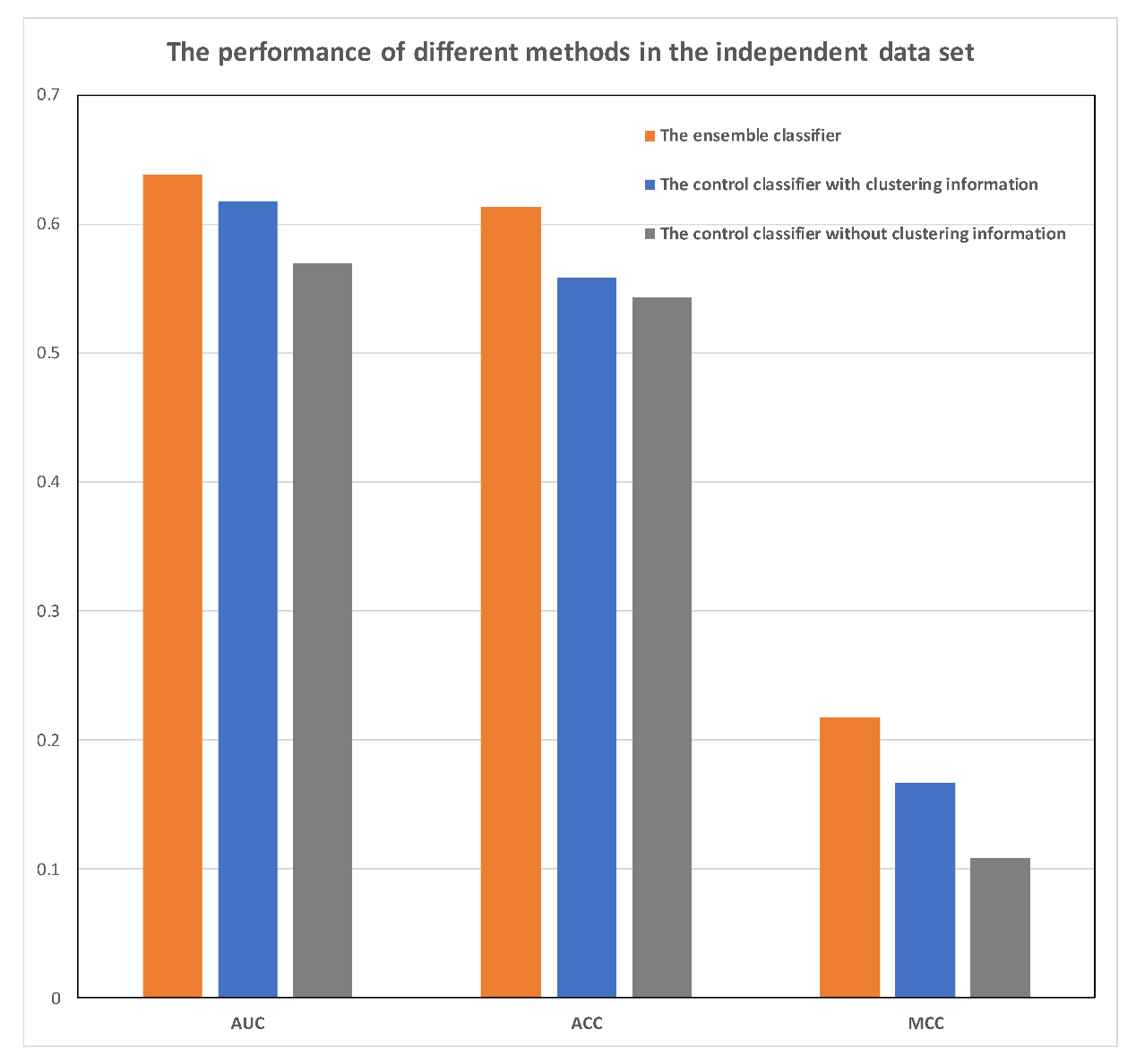


Figure S4: Comparison of the ensemble classifier with two control classifiers. These two control classifiers selected the features based on cox regression. The AUC, ACC and MCC of our classifier and two control classifiers on the independent data set. Because constructing the cox regression model was very time consuming, we used the five-fold cross validation to select the genes.


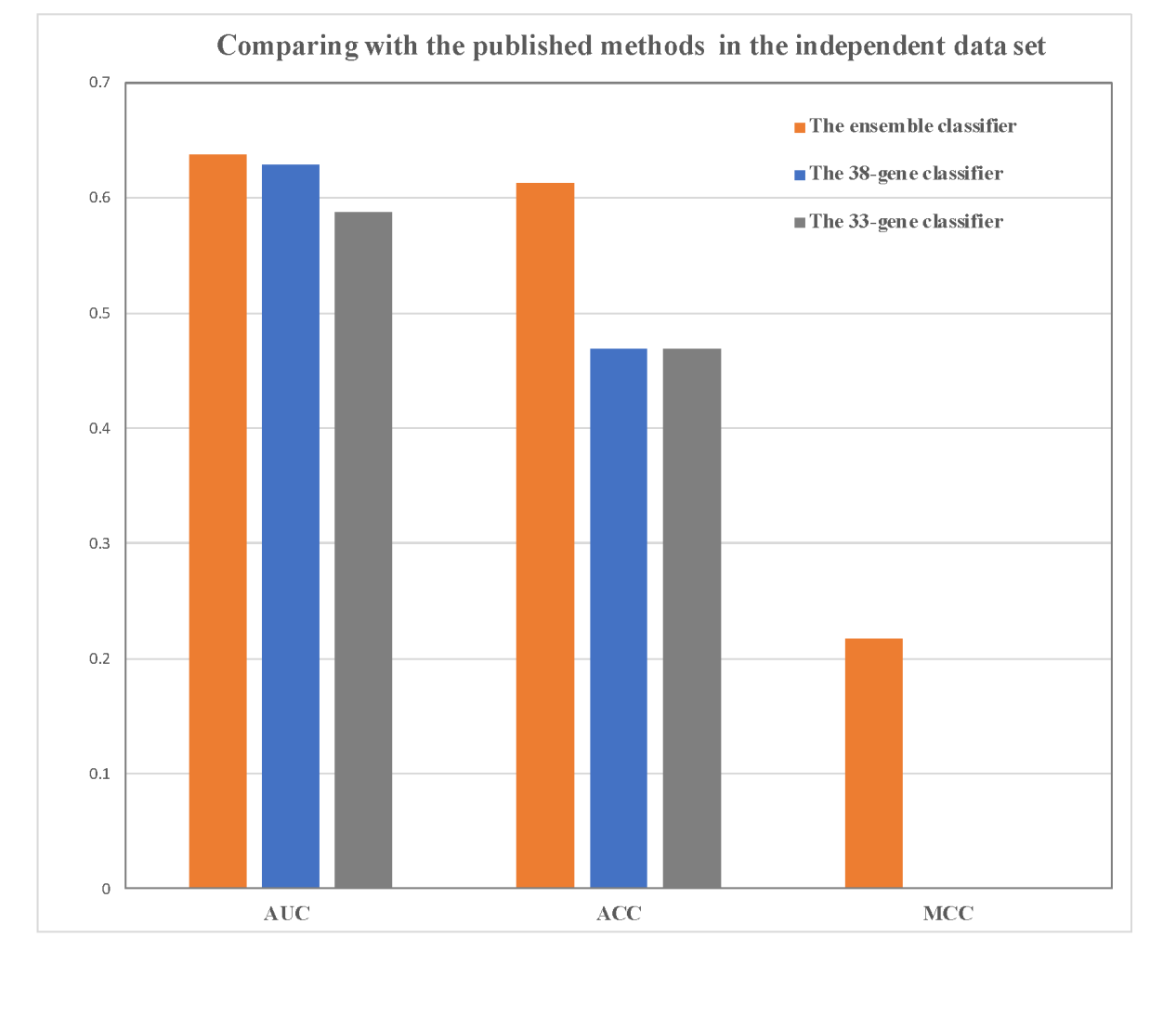


Figure S5: Comparison of ensemble classifier with two representative classifiers. The AUC, ACC and MCC of our classifier and two representative classifiers on the independent data set.
